# The effect of intermittent fasting on hedonic hunger: a pilot prospective cohort study based on Ramadan intermittent fasting

**DOI:** 10.3389/fnut.2025.1718105

**Published:** 2025-12-02

**Authors:** Halime Selen

**Affiliations:** 1 Department of Nutrition and Dietetics, Faculty of Health Sciences, Ağrı İbrahim Çeçen University, Ağrı, Türkiye; 2 Department of Nutrition and Dietetics, Faculty of Health Sciences, Mardin Artuklu University, Mardin, Türkiye

**Keywords:** body mass index, hedonic hunger, intermittent fasting, Ramadan intermittent fasting, weight loss

## Abstract

**Objective:**

This study aims to evaluate the changes in hedonic hunger (HH) during Ramadan intermittent fasting (RIF) and to investigate the role of HH as a factor influencing adherence to intermittent fasting (IF) regimens.

**Methods:**

This prospective cohort study was conducted between February 24 and March 29, 2025, with 122 participants aged between 20 and 25. The study data were collected through face-to-face interviews using a questionnaire that included sociodemographic information and the power of food scale (PFS), administered 1 week before the beginning of Ramadan, and during the first, middle period and final weeks of the month. The PFS comprises three subscales: food available, food present, and food tasted. In general, mean scores of the PFS and its subdimensions exceeding 2.5 are interpreted as indicating a high tendency toward HH.

**Results:**

During the first week of Ramadan, participants’ scores for food available (*p* = 0.016), food tasted (*p* = 0.002), and the PFS mean score (*p* = 0.048) were found to be significantly higher compared to the pre-Ramadan scores. However, no statistically significant differences were observed in the PFS mean and subscale scores between the pre-Ramadan period and the final week of RIF. Compared to the pre-Ramadan period, participants exhibited a mean reduction of 0.6 ± 1.51 kg in body weight (*p* < 0.001) and 0.3 ± 1.20 kg/m^2^ in BMI (*p* = 0.003) following RIF. A statistically significant but weak negative correlation was observed between changes in participants’ body weight and the scores of food available (*r* = −0.203, *p* = 0.025), food present (*r* = −0.340, *p* = *p* < 0.001), food tasted (*r* = −0.319, *p* < 0.001), and the PFS mean score (*r* = −0.323, *p* = *p* < 0.001). A statistically significant and weak negative correlation was found between changes in BMI and the scores of food present (*r* = −0.181, *p* = 0.046), food tasted (*r* = −0.216, *p* = 0.017), and the PFS mean score (*r* = −0.208, *p* = 0.021).

**Conclusion:**

This study suggests that higher levels of HH may be experienced at the onset of RIF practices; however, over time, individuals appear to adapt, returning to their pre-fasting levels of HH. Within the limited observation period of this study, the influence of RIF–a form of IF–on HH appears to be transient, suggesting that longer-term studies with larger sample sizes are needed to clarify its persistence.

## 1 Introduction

Intermittent fasting (IF), one of the increasingly popular dietary approaches in recent years, is a nutritional strategy that focuses more on when individuals eat rather than what they eat (1). IF practices are categorized into four main types: the 5:2 diet, alternate-day fasting, time-restricted eating, and religious fasting (2, 3).

The 5:2 diet is a nutritional strategy in which individuals eat without restriction for 5 days of the week, while on the remaining 2 days, energy intake is restricted to 800 kcal/day or less for men and 500 kcal/day or less for female (4, 5). Alternate-day fasting involves alternating between ad libitum eating days, during which individuals consume food freely, and fasting days, during which only 25% of the required energy intake is consumed. Each eating and fasting period typically lasts 24 h, although variations may occur (6). Time-restricted eating is a form of IF in which food intake is completely restricted for a 16-h period following an 8-h window during which eating is permitted (7). In time-restricted eating strategies, an eating window from 06:00 to 15:00 is defined as early time-restricted eating, whereas a window from 11:00 to 20:00 is referred to as late time-restricted eating (8). Religious fasting comprises various types of fasting practiced for spiritual or religious purposes. Among these, the Ramadan intermittent fasting (RIF), observed by Muslim communities, involves complete abstinence from food and drink from dawn until sunset during the month of Ramadan, which occurs at different times each year (9). The daily fasting duration during the 29–30 days of Ramadan varies according to seasonal and geographical differences. Although the most commonly practiced form of IF today is time-restricted eating, which involves 16 h of fasting followed by an 8-h eating window, RIF-based studies are frequently encountered in the literature due to the relative ease of study design.

Current evidence indicates that IF regimens promote weight loss, improve oxidative stress and inflammatory markers, enhance glycometabolic parameters, reduce cardiometabolic risks, confer protection against non-alcoholic fatty liver disease, lower the risk of cardiovascular disease, ameliorate components of metabolic syndrome, and modulate the gut microbiota (10–17). These findings contribute to the growing popularity of IF practices. However, how IF affects individuals’ hedonic hunger (HH) and food intake remains largely unclear.

In humans, food intake and hunger are primarily regulated by two mechanisms: homeostatic and hedonic (18). While the homeostatic mechanism is influenced by physiological factors such as depleted energy stores and low blood glucose levels, the hedonic mechanism refers to the desire to eat irrespective of energy needs, often driven by the appeal of palatable foods (19, 20). However, the hedonic drive can override the homeostatic mechanism, leading to food consumption even in the presence of sufficient energy availability (19, 21). Although studies on this subject are limited, research conducted both in Türkiye and internationally indicates that HH has a considerable prevalence across different populations. In a study conducted among female university students in Indonesia, 31.3% of participants were reported to have a high tendency toward HH (22). Studies carried out in various populations in Türkiye have shown that the tendency toward HH ranges between 50 and 65%, and that overweight or obese individuals exhibit a higher inclination toward HH (23–25). This condition appears to be an important factor that hinders dietary adherence, particularly among individuals attempting to lose body weight.

IF practices can influence not only energy balance and metabolic processes but also hedonic drives and reward-based eating behaviors through the mesocorticolimbic dopamine system (26). A study using functional magnetic resonance imaging to investigate the effects of nutritional state on brain reward systems demonstrated that fasting can enhance sensitivity in reward-related regions such as the ventral striatum, amygdala, anterior insula, and orbitofrontal cortex, thereby modulating dopaminergic responses (27). The same study reported that during fasting periods, the subjective appeal of high-calorie foods increased more than that of low-calorie foods (27). Conversely, some authors argue that regular IF may normalize brain reward system function and promote more controlled eating behaviors. In a study by Breit et al., participants following a 4:3 IF regimen exhibited a significant reduction in binge and uncontrolled eating behaviors after 12 months (28). A study evaluating the potential role of IF on food reward responses suggested that IF protocols may modulate reward system-dependent eating behaviors, particularly affecting dopaminergic activity, neurotrophic factors, and reward-related responses to food (26). In the context of eating disorders, neuroimaging and pharmacological studies have shown functional alterations in reward pathways and dopaminergic differences, especially in conditions such as binge eating and bulimia nervosa (29–31). The effects of IF on these populations remain under investigation; while some studies suggest it may be safe and beneficial, others report that it could influence reward responses or eating behaviors in certain individuals. In a study assessing young adults, those engaging in IF reported higher eating disorder psychopathology, and a history of IF was associated with increased binge eating (32). In contrast, an 8-week randomized controlled trial in overweight women found no significant differences between IF and calorie-restricted groups in terms of eating behaviors, perceived hunger, or emotional eating (33). Therefore, the effects of IF on hedonic drives and eating disorders are not yet conclusive and may vary depending on individual differences and the specific fasting regimen.

Therefore, the aim of the present study is to evaluate the effect of RIF, a form of IF recognized as a body weight management strategy, on HH, and to explore the role of HH as a factor influencing the adherence to IF regimens.

## 2 Materials and methods

### 2.1 Study period, design, setting, population, and sample

This research is a prospective cohort study conducted between February 24 and March 29, 2025. The study population consisted of a total of 147 actively enrolled 3rd- and 4th-year students in the Department of Nutrition and Dietetics at the Faculty of Health Sciences, Ağrı İbrahim Çeçen University, located in Ağrı, Türkiye. The study sample included 122 students aged between 20 and 25 who had no physician-diagnosed chronic or metabolic diseases, were not taking any medications, and voluntarily fasted during the month of Ramadan (March 1–29, 2025), excluding menstruation weeks for female participants. The study data were collected across four periods: before Ramadan (February 24–26, 2025), and during the first week (March 5–7, 2025), middle period (March 14–16, 2025), and final week of Ramadan (March 27–29, 2025). During the study period, the minimum daily fasting duration was 12 h and 54 min on the shortest day (March 1, 2025) and 14 h and 10 min on the longest day (March 29, 2025).

Prior to data collection, participants were presented with a written Informed Consent Form, one copy of which was given to each participant for signing. All data were collected through face-to-face interviews, during which the researcher administered the questionnaires directly. All data collection procedures were conducted solely by the researcher, a nutrition specialist, to ensure consistency and standardization in data gathering.

### 2.2 Data collection tools

The study data were collected through face-to-face interviews using a questionnaire prepared by the researcher based on a literature review, which included sociodemographic information, anthropometric measurements and the power of food scale (PFS). The questionnaire was conducted in Turkish and administered in a paper-based format.

#### 2.2.1 Sociodemographic information questionnaire

This form included questions regarding participants’ age, sex, academic year, marital status, place of residence, working status, income level, smoking habits, and alcohol consumption.

#### 2.2.2 Anthropometric measurements questionnaire

This form included questions regarding participants’ height, and body weight. One week before the start of Ramadan (February 24–26, 2025), participants’ height (m) was measured using a digital Ultrasonic Harpenden Stadiometer (ADE/Hamburg MZ10020) with 0.1 cm precision. Body weight (kg) was measured 1 week before the start of Ramadan (February 24–26, 2025) and on the final week of Ramadan (March 27–29, 2025) after a 12-h fasting period, using a precision scale (ALTUS AL 808 SM) with 0.1 kg sensitivity. Based on these measurements, body mass index (BMI = kg/m^2^) values were calculated for each participant and classified according to the World Health Organization (WHO) criteria as follows: <18.5 kg/m^2^ “Underweight,” 18.5–24.9 kg/m^2^ “Normal,” 25.0–29.9 kg/m^2^ “Overweight,” and ≥30 kg/m^2^ “Obese” (34).

#### 2.2.3 Power of food scale (PFS)

The PFS, was originally developed by Lowe et al. (35), and its Turkish validity and reliability were established by Akçil Ok and Hayzaran (36). The PFS consists of 15 items rated on a 5-point Likert-type scale (1 = strongly disagree, 5 = strongly agree). The mean PFS score is calculated by dividing the total score by the number of items. The same scoring system is applied to the subscales. The scale consists of three subscales: food available, food present, and food tasted. In general, mean scores of the PFS and its subdimensions exceeding 2.5 are interpreted as indicating a high tendency toward HH (36).

##### 2.2.3.1 Food available

This subscale, which includes items 1, 2, 5, 10, 11, and 13, is based on the hypothetical presence of palatable foods in the environment. It captures individuals’ responses to a food context in which foods are always mentally available but not physically present.

##### 2.2.3.2 Food present

This factor, which includes items 3, 4, 6, and 7, refers to situations where palatable foods are physically present in the environment but have not yet been tasted.

##### 2.2.3.3 Food tasted

This factor, which includes items 8, 9, 12, 14, and 15, describes situations in which palatable foods have been tasted but not fully consumed.

### 2.3 Ethical considerations

This study was approved by the Scientific Research Ethics Committee of Ağrı İbrahim Çeçen University (Ethical Approval Number: 41, dated January 30, 2025). The study was conducted in accordance with the Declaration of Helsinki.

### 2.4 Statistical analysis

The data were initially analyzed using DataBeeg 1.0, an AI-powered software developed in Türkiye in 2023 that supports qualitative, quantitative, and mixed-method analyses. For methodological validation, the results were subsequently cross-checked with SPSS software version 27.0 (IBM Corp., Armonk, NY, USA). The findings obtained from both software packages were convergent and mutually supportive. Descriptive statistical methods (frequency, percentage, mean, and standard deviation) were used for data evaluation. The normality of the data distribution was examined using Q-Q plots (37), and normal distribution was considered present if skewness and kurtosis values were within the ±3 range (38). For data with a normal distribution, the paired samples t-test was used for comparisons between two dependent time points, and repeated measures ANOVA was applied for comparisons involving more than two dependent time points (before Ramadan and during the first week, middle period, and final week of Ramadan). In cases where significant differences were found in the ANOVA results, pairwise comparisons were conducted using the Bonferroni correction to identify the source of the difference. Pearson correlation analysis was conducted to assess the relationship between two numerical variables that satisfy the assumption of normality. A value of *p* < 0.05 was considered statistically significant.

## 3 Results

The baseline characteristics of the study participants, assessed during the period before Ramadan, are presented in Table 1. The participants had a mean age of 22.54 ± 1.22 years, and 80.3% were female. The majority of the participants were single (95.9%), living in student dormitory (76.2%), not working (91.8%), of average-income status (70.5%), non-smokers (82.0%), did not consume alcohol (93.4%). Additionally, 68.9% had a normal BMI, and most exhibited a high tendency toward HH (86.9%).

**Table 1:** Baseline characteristics of the study population taken before Ramadan (*n* = 122).

Variables		*n*	%
Age (years) ( $\bar{X}$ ±SD = 22.54 ± 1.22)	22 years and under	67	54.9
	Over 22 years	55	45.1
Sex	Female	98	80.3
	Male	24	19.7
Academic year	Bachelor’s degree 3rd year	72	59.0
	Bachelor’s degree 4th year	50	41.0
Marial status	Married	5	4.1
	Single	117	95.9
Place of residence	Student dormitory	93	76.2
	Family home	21	17.2
	Private home	8	6.6
Working status	Working	10	8.2
	Not working	112	91.8
Income status	Good	27	22.1
	Average	86	70.5
	Poor	9	7.4
Smoking status	Yes	22	18.0
	No	100	82.0
Alcohol consumption	Yes	8	6.6
	No	114	93.4
BMI classification	Underweight	12	9.8
	Normal	84	68.9
	Overweight	20	16.4
	Obese	6	4.9
PFS and subdimensions	Food available (≤2.5)	56	45.9
	Food available (>2.5)	66	54.1
	Food present (≤2.5)	22	18
	Food present (>2.5)	100	82
	Food tasted (≤2.5)	7	5.7
	Food tasted (>2.5)	115	94.3
	PFS mean (≤2.5)	16	13.1
	PFS mean (>2.5)	106	86.9

Descriptive statistics were expressed as frequency (*n*) and percentage (%). BMI, body mass index; PFS, power of food scale.

The comparison of participants’ body weight and BMI values before and after Ramadan is presented in Table 2. A statistically significant decrease was observed in both body weight and BMI values following Ramadan (*p* < 0.05). On average, participants lost 0.6 ± 1.51 kg in weight (*p* = 0.000), and their BMI values decreased by 0.3 ± 1.20 kg/m^2^ (*p* = 0.003).

**Table 2:** Comparison of participants’ anthropometric measurement before and after Ramadan (*n* = 122).

Variables	Measurement time	Med	$\bar{X}$	SD	p*
Body weight	Before Ramadan	60.50	63.27	12.44	**0.000** ^**t**^
	After Ramadan	60.85	62.68	11.93	
BMI values (kg/m^2^)	Before Ramadan	22.65	22.96	3.41	**0.003** ^**t**^
	After Ramadan	22.50	22.63	3.30	

^t^Paired samples *t*-test. *p < 0.05. BMI, body mass index. Values in bold in the tables indicate “*p < 0.05”.

The comparison of participants’ PFS mean and subscale scores before Ramadan and during the first, middle period, and final weeks of Ramadan is presented in Table 3. Accordingly, the scores for food available (*p* = 0.016), food tasted (*p* = 0.002), and the PFS mean score (*p* = 0.048) measured during the first week of Ramadan were found to be significantly higher than the pre-Ramadan scores. However, no statistically significant differences were observed between the pre-Ramadan and final week scores in the PFS mean and subscale scores.

**Table 3:** Comparison of participants’ PFS mean and subscale scores before Ramadan and during the first week, middle period, and final week of Ramadan (*n* = 122).

Variables	Measurement time	Med	$\bar{X}$	SD	p*	Bonferroni
Food available	Before Ramadan (1)	2.67	2.68	0.67	**0.016** ^**A**^	**2 > 1**
	First week of Ramadan (2)	2.83	2.87	0.72		
	Middle period of Ramadan (3)	2.83	2.80	0.75		
	Final week of Ramadan (4)	2.67	2.75	0.75		
Food present	Before Ramadan (1)	3.25	3.29	0.71	0.122^A^	–
	First week of Ramadan (2)	3.25	3.23	0.66		
	Middle period of Ramadan (3)	3.00	3.15	0.71		
	Final week of Ramadan (4)	3.25	3.16	0.78		
Food tasted	Before Ramadan (1)	3.40	3.31	0.57	**0.002** ^**A**^	**2 > 1**
	First week of Ramadan (2)	3.60	3.52	0.59		
	Middle period of Ramadan (3)	3.60	3.41	0.65		
	Final week of Ramadan (4)	3.60	3.39	0.71		
PFS mean	Before Ramadan (1)	3.00	3.05	0.52	**0.048** ^**A**^	**2 > 1**
	First week of Ramadan (2)	3.20	3.18	0.53		
	Middle period of Ramadan (3)	3.07	3.10	0.59		
	Final week of Ramadan (4)	3.07	3.07	0.64		

^A^ANOVA test. *p < 0.05. BMI, body mass index; PFS, power of food scale. Values in bold in the tables indicate “*p < 0.05”.

The relationship between changes in participants’ body weight and BMI and HH is presented in Table 4. A statistically significant but weak negative correlation was found between changes in body weight and the scores of food available (*r* = −0.203, *p* = 0.025), food present (*r* = −0.340, *p* = 0.000), food tasted (*r* = −0.319, *p* = 0.000), and the PFS mean score (*r* = −0.323, *p* = 0.000). Similarly, changes in BMI were found to have a statistically significant but weak negative correlation with food present (*r* = −0.181, *p* = 0.046), food tasted (*r* = −0.216, *p* = 0.017), and the PFS mean score (*r* = −0.208, *p* = 0.021).

**Table 4:** The relationship between changes in participants’ body weight and BMI and hedonic hunger (*n* = 122).

Variables	Body weight change		BMI change	
	*r*	p*	*r*	p*
Food available	−0.203	**0.025**	−0.148	0.103
Food present	−0.340	**0.000**	−0.181	**0.046**
Food tasted	−0.319	**0.000**	−0.216	**0.017**
PFS mean	−0.323	**0.000**	0.208	**0.021**

*r*: Pearson correlation coefficient. *p < 0.05. BMI, body mass index; PFS, power of food scale. Values in bold in the tables indicate “*p < 0.05”.

## 4 Discussion

There is substantial evidence supporting the health-promoting effects of IF (39). However, one of the major factors that may hinder adherence to IF is the experience of hunger, which can lead individuals to discontinue the practice (40). This study aimed to evaluate the effect of RIF on HH.

Participants in the present study were found to have lost weight by the end of Ramadan. Although participants’ dietary intake and physical activity levels were not assessed in this study, the findings were generally consistent with the existing literature (1). A meta-analysis of 85 studies involving individuals aged 16 and above demonstrated that IF significantly reduced body weight, although the degree of change may vary by country and season (10). Another meta-analysis including 65 studies conducted among individuals aged 18 and over, which compared the effects of RIF and other forms of IF on body composition, reported that both types of fasting were effective in reducing body weight (41). These findings suggested that IF could serve as an effective dietary strategy for weight loss, independent of energy intake and physical activity.

In the present study, increases were observed only during the first week of Ramadan in participants’ scores for food available, food tasted and PFS maen, all of which are associated with HH. By the final week of Ramadan, these scores had returned to levels comparable to those recorded before the fasting period. This pattern suggests that HH tends to rise during the early phase of IF but diminishes over time as individuals adapt. Similarly, in a large-scale observational study involving 1,422 participants, individuals reported experiencing stronger feelings of hunger and food cravings at the beginning of IF regimens (42). In a study comparing individuals following a 5:2 diet with those undergoing continuous energy restriction, both groups showed a reduction in hunger levels after a 12-week intervention, with no significant differences between them (43). However, in a study by Sundfør et al. (2018), individuals who followed the 5:2 diet for 1 year reported experiencing greater hunger compared to those on continuous energy restriction (44). Similarly, a study conducted in Türkiye found a significant increase in “food tasted” scores—an indicator of HH—when comparing participants’ scores before and at the end of RIF (45). A meta-analysis evaluating 17 randomized controlled trials found no significant differences between IF and continuous energy restriction diets in terms of hunger, satiety, food cravings, or food intake (46). These findings suggest that although hunger may increase during the initial phase of IF, it tends to diminish over time, indicating that one of the key barriers to adherence gradually weakens. However, consistent with the present study’s findings, existing literature did not provide strong evidence that IF led to a substantial reduction in appetite or HH levels overall. While the present study does not propose a potential mechanism for HH in the context of RIF, a recent review article argues that IF reduces cravings for unhealthy foods and modulates homeostatic responses in the hypothalamus as well as dopaminergic activity within the reward system (26).

The study found a negative relationship between changes in participants’ body weight and BMI and their levels of HH and related subscales. This suggests that weight loss may be associated with a reduction in HH. Previous studies involving different age groups have shown that individuals with obesity tend to have higher HH scores (47–49). However, there is limited research examining how HH scores change in response to weight loss. A similar study conducted with individuals who underwent bariatric surgery reported a decrease in HH scores following weight loss (50). In a study involving 283 participants with a BMI ranging from 27 to 45 kg/m^2^ who took part in a 12-month behavioral weight loss program, reductions in HH were found to be associated with weight loss (51). Similarly, in a study evaluating 111 adults with a mean BMI of 31.5 ± 2.7 kg/m^2^ who participated in a 12-week weight loss program, decreases in HH were associated with greater weight loss success (52). Conversely, another study reported that a full-point increase in the PFS domain score was associated with a 1.6–2.3 times greater likelihood of being obese (53). These findings, which align with the results of the present study, highlight the importance of understanding the bidirectional relationship between obesity and HH. Nonetheless, consistent with previous literature, the primary limitation of the present study is the lack of assessment of participants’ energy intake, macronutrient and micronutrient consumption, and physical activity levels. Interpreting directly the relationship between HH and weight loss without accounting for these factors would be inappropriate. Observed weight loss may have already been driven by energy restriction, which could have directly impacted HH. Therefore, to elucidate this relationship more comprehensively, randomized controlled trials focusing solely on time-restricted eating protocols, independent of energy restriction, are warranted.

### 4.1 Limitations of the study

The main limitations of this study are that the sample consisted of a relatively small and age-limited group of university students, and that the participants’ dietary intake and physical activity levels before and during the fasting period were not assessed. It was also evident that a one-month follow-up was a relatively short duration for fully understanding the effects of IF on HH, highlighting the need for longer-term studies. Furthermore, the relatively homogeneous (university students, aged 20–25, predominantly female, nutrition/dietetics majors), nature of the sample limits the applicability of the study results to individuals with different health conditions (diabetes mellitus, hypertension, hyperlipidemia, cardiovascular diseases, etc.), the elderly, or different broad populations with a male majority. Additionaly, although the PFS is widely used to assess HH, it may not fully capture all dimensions of hedonic eating. Future studies in this field could benefit from combining the PFS with additional tools such as the Palatable Eating Motives Scale (PEMS) to provide a more comprehensive evaluation.

### 4.2 Rationale of the study

Considering the limited number of studies in the literature examining the effect of IF on HH, the present study offered valuable insights into the feasibility of such dietary practices in light of its findings and significance. Nevertheless, there remains a need for longitudinal studies with larger sample sizes and extended follow-up periods to better evaluate the impact of IF on HH.

## 5 Conclusion

IF has become a prominent topic of research in recent years and is generally recognized as a dietary approach with potential health benefits. However, the prolonged fasting periods involved may lead to intense feelings of hunger, which can act as a barrier to adherence. This study suggested that individuals might experience elevated levels of HH at the beginning of RIF, but tend to adapt over time, with levels returning to those observed prior to fasting. Within the limited observation period of this study, the influence of RIF–a form of IF–on HH appears to be transient, suggesting that longer-term studies with larger sample sizes are needed to clarify its persistence.

## Data Availability

The raw data supporting the conclusions of this article will be made available by the authors, without undue reservation.
